# Prevalence of SARS-CoV-2, Verona, Italy, April–May 2020

**DOI:** 10.3201/eid2701.202740

**Published:** 2021-01

**Authors:** Massimo Guerriero, Zeno Bisoffi, Albino Poli, Claudio Micheletto, Antonio Conti, Carlo Pomari

**Affiliations:** Istituto di Ricovero e Cura a Carattere Scientifico Ospedale Sacro Cuore Don Calabria, Negrar, Italy (M. Guerriero, Z. Bisoffi, A. Conti, C. Pomari);; Università di Verona, Verona, Italy (Z. Bisoffi, A. Poli);; Azienda Ospedaliera Universitaria di Verona, Verona (C. Micheletto)

**Keywords:** SARS-CoV-2, COVID-19, IgG, RT-PCR, random sample survey, latent class analysis, respiratory infections, severe acute respiratory syndrome coronavirus 2, 2019 novel coronavirus disease, coronavirus disease, zoonoses, viruses, coronavirus, Italy

## Abstract

We used random sampling to estimate the prevalence of severe acute respiratory syndrome coronavirus 2 infection in Verona, Italy. Of 1,515 participants, 2.6% tested positive by serologic assay and 0.7% by reverse transcription PCR. We used latent class analysis to estimate a 3.0% probability of infection and 2.0% death rate.

On May 25, 2020, Italy had the third highest number of cases and the second highest number of deaths in Europe caused by the novel betacoronavirus severe acute respiratory syndrome coronavirus 2 (SARS-CoV-2) (*1*) as part of the ongoing pandemic of coronavirus disease (COVID-19). The continuing spread of infection and the resulting strain on healthcare systems has made the identification of asymptomatic persons crucial to limiting transmission (*2*–*6*). We conducted a cross-sectional study on a representative sample of the general population to estimate the prevalence and death rate of SARS-CoV-2 infection in Verona, Italy.

## The Study

We estimated the prevalence of active or past SARS-CoV-2 infection among the population of Verona among randomly selected participants >10 years of age. This investigation was an observational, cross-sectional study approved by the Ethics Committee of Verona and Rovigo provinces on April 15, 2020 (internal protocol no. 2641CESC), in compliance with the Strengthening the Reporting of Observational Studies in Epidemiology guidelines (*7*).

According to Verona’s municipal register, 235,034 persons >10 years of age lived in Verona on January 1, 2020 (*8*). We used systematic random sampling to compile a list of potential participants. Because the prevalence of asymptomatic SARS-CoV-2 infection in Italy had previously been estimated at 10.0% (*9*,*10*), we decided to randomly sample 1,527 participants, resulting in a standard error of <1.5%. We predicted a dropout rate of 35% and accordingly mailed invitations to 2,061 potential participants. We selected the first sample using a random starting point; for subsequent samples, we used a sampling interval calculated by dividing the population size by the desired sample size (235,034/2,061 = 114).

We collected data from April 24 through May 8, 2020. We required a parent’s or guardian’s consent for participants <18 years of age. All participants gave their informed consent. Participants first completed a phone interview about COVID-19 symptoms within the previous 15 days. Specialized staff at Istituto di Ricovero e Cura a Carattere Scientifico then collected blood and nasopharyngeal swab samples from each participant. These staff extracted total RNA from nasopharyngeal swab samples using a MagnaPure LC.2 instrument and MagNA Pure LC RNA Isolation Kit (Roche Molecular Systems Inc., https://lifescience.roche.com), according to the manufacturer’s instructions. We analyzed the eluted RNA by reverse transcription PCR (RT-PCR) to detect the presence of active infections (*11*). We analyzed serum samples for IgG against SARS-CoV-2 by serologic assay (Abbott, https://www.abbott.com) to detect previous infections. Experienced laboratory personnel conducted each test independently and blindly.

Because neither assay has perfect sensitivity, we used latent class analysis (LCA) to estimate the prevalence of SARS-CoV-2 infection. LCA models were based on SARS-CoV-2 test results and selected clinical variables (*12*). We interpreted the outcomes as the probability that a given person was (or had been) infected (*13*). We reported all parameters and estimations with 95% CIs. We adjusted statistical models and estimations for covariates.

A total of 1,515 persons participated in the study (Figure). We found no significant difference in sex proportions between the general population (53% female) and the study sample (54% female). The mean age of all participants was 52.1 (SD = 20.0) years in the general population and 49.1 (SD = 22.2) years in the study sample. We summarized demographic and clinical data using descriptive statistics and measures of variability and precision (Table 1). Of 1,515 participants, 9 (0.6%) tested positive for SARS-CoV-2 RNA but negative for IgG against SARS-CoV-2, 40 (2.6%) tested negative for viral RNA but positive for IgG, and 1,465 (96.7%) tested negative for both indicators. Only 1 participant tested positive for RNA and IgG. Participants who tested negative for viral RNA but positive for IgG reported symptoms such as anosmia (39.5%), temperature >37.5°C (30.8%), fatigue (35.9%), and persistent cough (28.2%). Less than 2% of participants who tested negative by both tests reported symptoms.

**Figure:**
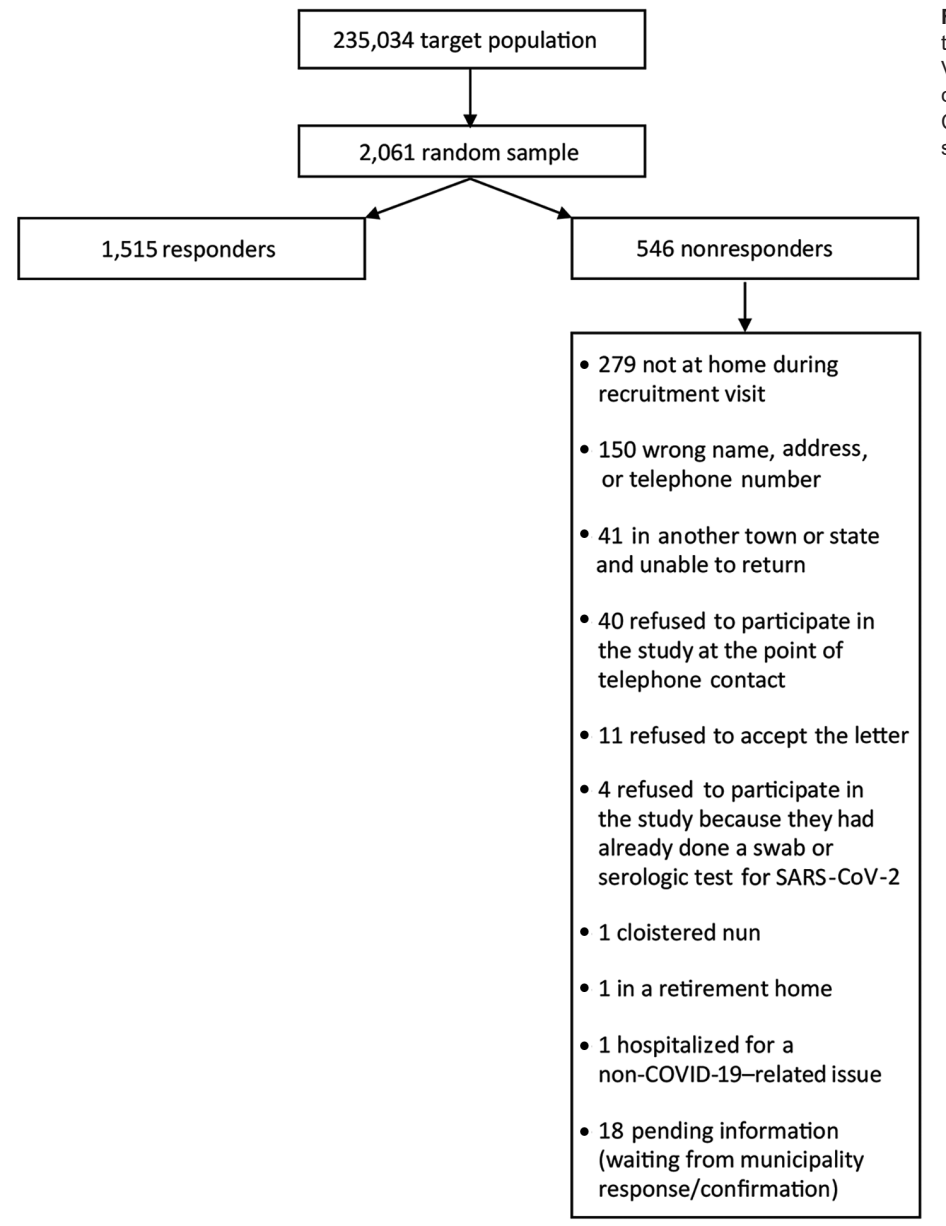
Flowchart of participant testing for SARS-CoV-2 infection, Verona, Italy, 2020. COVID-19, coronavirus disease; SARS-CoV-2, severe acute respiratory syndrome coronavirus 2.

**Table 1:** Demographic and clinical characteristics of participants tested for SARS-CoV-2, Verona, Italy, 2020*

Characteristic	Total	RNA-positive	RNA-negative/IgG-positive	RNA-negative/IgG-negative	p value†	Missing values
Total, no. (%)	1,515 (100.0)	10 (0.7)	40 (2.6)	1465 (96.7)	NA	0
Mean age, y (SD)	52.1 (20.0)	53.4 (15.5)	47.3 (17.6)	52.2 (20.1)	0.31	0
Sex, no.						
M	699	3	17	679	0.57	0
F	816	7	23	786		0
SARS-CoV-2 symptoms, %						
Anosmia	2.4	10.0	39.5	1.4	<0.01	3.9
Conjunctivitis	8.0	0	12.8	7.9	0.38	3.1
Temperature >37.5°C	2.7	10.0	30.8	1.8	<0.01	3.1
Dry cough or phlegm	9.3	0	28.2	8.9	<0.01	3.0
General muscle pain	5.4	10.0	23.1	4.9	<0.01	3.2
Fatigue	8.3	10.0	35.9	7.5	<0.01	3.1
Headache	10.4	10.0	15.4	10.2	0.48	3.0
Sore throat	7.6	0	7.7	7.6	1.00	3.2
Chills	4.4	10.0	10.5	3.9	<0.01	3.0
Diarrhea	7.9	20.0	13.2	7.7	0.09	3.2
Dyspnea	3.5	10.0	18.0	3.0	<0.01	3.2
Nausea/vomiting	2.6	10.0	10.5	2.3	<0.01	3.1

*Values are no. (%) except as indicated. NA, not applicable; SARS-CoV-2, severe acute respiratory syndrome coronavirus 2. †Mean difference in age tested with analysis of variance. All other p values calculated by Fisher’s exact test.

We used a backward stepwise multinomial multivariate logistic regression model to compare selected COVID-19 symptoms (i.e., anosmia, dyspnea, diarrhea, and fever) in the RNA-positive and RNA-negative/IgG-positive groups with the RNA-negative/IgG-negative group. Fever and anosmia were each significantly associated with belonging to the RNA-negative/IgG-positive group (p<0.01) but not the RNA-positive group.

We used LCA to estimate the prevalence of infection considering the results of RT-PCR, the serologic assay, and the symptoms selected by stepwise regression. The estimated probability of belonging to class 1 (uninfected) was 0.97 and class 2 (infected) was 0.03 (Table 2).

**Table 2:** Latent class analysis of SARS-CoV-2 infection, Verona, Italy, 2020

Factor	Probability % (95% CI)			% (95% CI)			
	Class 1 (SARS-CoV-2–negative)	Class 2 (SARS-CoV-2–positive)		Sensitivity	Specificity	Positive predictive value	Negative predictive value
SARS-CoV-2 IgG	1.1 (0.6–2.0)	53.5 (30.8–74.8)		53.5 (51.0–53.0)	98.9 (98.4–99.4)	60.1 (57.6–62.5)	98.6 (98.0–99.2)
SARS-CoV-2 RNA	0.5 (0.2–1.1)	6.1 (1.6–20.9)		6.1 (4.9–5.7)	99.5 (99.1–99.9)	27.4 (25.1–29.6)	97.2 (96.3–98.0)
Anosmia*	0.8 (0.03–1.8)	54.5 (33.2–74.2)		54.5 (52.0–54.1)	99.2 (98.8–99.6)	67.8 (65.5–70.2)	98.6 (98.0–99.2)
Diarrhea*	7.0 (5.7–8.6)	37.2 (21.6–55.9)		37.2 (34.8–35.9)	93.0 (91.7–94.3)	14.1 (12.4–15.9)	98.0 (97.2–98.7)
Fever*	1.3 (0.8–2.2)	45.8 (26.3–66.7)		45.8 (43.3–45.2)	98.7 (98.1–99.3)	52.1 (49.6–54.7)	98.3 (97.7–99.0)
Dyspnea*	2.6 (1.8–3.6)	32.3 (17.9−51.0)		32.3 (29.9–31.5)	97.4 (96.6–98.2)	27.8 (25.5–30.0)	97.9 (97.2–98.6)

*The clinical variables used in the latent class analysis were selected by backward stepwise multinomial logistic regression with a significance level for removal of 0.2.

As of May 25, 2020, Verona had 1,528 cumulative patients in whom SARS-CoV-2 infection was diagnosed, including 144 who had died, indicating a 9.4% death rate (*14*). Verona was the province in Veneto with the most cases and deaths caused by SARS-CoV-2 (*15*). Our LCA estimated a prevalence of 3.0%, suggesting 7,051 cumulative cases (4.6 times higher than the official count). These estimates suggest that 144 reported deaths would indicate a 2.0% death rate. According to the crude rates, the 50 SARS-CoV-2–positive participants in our study would account for 3.3% of the total study population. Applying this percentage to the whole population of Verona would indicate 7,756 cases and a 1.9% death rate.

Of the 10 RNA-positive participants, only 1 tested positive by serologic assay. This finding raises concerns about the current screening policy of 2-step testing, which comprises a serologic assay and, if the assay results are positive, PCR. Given the economic costs associated with testing, officials should carefully advise the public on all testing options.

Our study has a few limitations. Because participation was voluntary, our study might have been influenced by selection bias (Figure). Also, LCA might have underestimated the accuracy of both diagnostic tests. For example, considering past and active infections together might have reduced test sensitivity. Furthermore, the PCR assay did not have 100% specificity, as is usually assumed (A.N. Cohen, unpub. data, https://www.medrxiv.org/content/10.1101/2020.04.26.20080911v4). The model might have also underestimated the specificity of the serologic assay. However, the crude rates estimate a prevalence only slightly higher, and the death rate only slightly lower, than predicted by our model.

## Conclusions

Our study estimated the prevalence of SARS-CoV-2 infection in Verona using a random sample of its population. Similar studies are currently underway on a larger scale. The results will estimate the true circulation of SARS-CoV-2, better approximate the death rate, and inform infection containment and management. Our study provides a clear picture of the circulation of SARS-CoV-2 infection in the general population of a city and an estimation of the true death rate caused by the infection. The results also suggest that 2-step testing might not detect all active infections. We are currently organizing phase 2 of our study, during which we will conduct follow-up serologic testing on all PCR-positive and PCR-negative/IgG-positive participants, enabling the evaluation of any antibody seroconversion, negativization, or change in titer.
